# Targeting the Cargo Receptor TMED9 as a Therapeutic Strategy Against Brain Tumors

**DOI:** 10.3390/cells14110772

**Published:** 2025-05-23

**Authors:** Alaa Daoud Sarsour, Sara Kinstlinger, Rephael Nizar, Naama Amos, Narkis Arbeli, Gila Kazimirsky, Irena Bronshtein-Berger, Iris Fried, Ron Unger, Chaya Brodie, Moran Dvela-Levitt

**Affiliations:** 1 The Mina and Everard Goodman Faculty of Life Sciences, Bar-Ilan University, Ramat Gan 52900, Israel; daoudsa2@biu.ac.il (A.D.S.); sara.kinstlinger@gmail.com (S.K.); rephael92@gmail.com (R.N.); amosnaama@gmail.com (N.A.); kisi96@gmail.com (N.A.); gilakazimirsky@gmail.com (G.K.); irena.bronshtein@biu.ac.il (I.B.-B.); ron.unger@biu.ac.il (R.U.); 2 Institute of Nanotechnology and Advanced Materials (BINA), Bar-Ilan University, Ramat Gan 52900, Israel; 3 Pediatric Hematology Oncology Unit, Shaare Zedek Hospital, Jerusalem 9103102, Israel; ishonet@gmail.com

**Keywords:** glioblastoma, glioma stem cells, TMED, p24, DIPG

## Abstract

Glioblastoma is one of the most aggressive and lethal forms of brain cancer, with limited therapeutic options and poor patient prognosis. Recent research has identified the TMED family of proteins as key regulators of tumor progression and aggressiveness across multiple cancer types. TMED members are cargo receptors expressed within the early secretory pathway and involved in bidirectional traffic of various proteins including EGFR, TGF-ɑ and WNT. In this study, we explored the therapeutic potential of genetic and pharmacologic inhibition of the cargo receptor TMED9 in glial tumor models. Our findings demonstrate that TMED9 expression is upregulated in glioma and that this upregulation is associated with poor patient survival. Using patient-derived glioma tumor cells, we demonstrate that TMED9 is highly expressed in the cancer stem cell population and that this upregulation promotes the cells’ self-renewal and migration. This is the first time, to the best of our knowledge, that TMED9 has been shown to play a major role in the function and tumorigenesis of brain tumor cancer stem cells. BRD4780, a small molecule that targets TMED9, effectively reduced TMED9 abundance, resulting in decreased viability, migration and stemness of patient-derived glioma stem cells. Moreover, BRD4780 mitigated the proliferation and migration of differentiated glioma tumor cells. When applied together with temozolomide, an established glioblastoma treatment, BRD4780 elicited an enhanced anti-tumor response. Lastly, to demonstrate the broad applicability of our findings, we targeted TMED9 in pediatric glioma cells and showed efficient inhibition of various oncogenic functions. Collectively, our study identifies TMED9 inhibition as a promising therapeutic approach that impairs the tumorigenesis and aggressiveness of brain tumors, with high efficacy against the tumor stem cell population. The effectiveness of TMED9 targeting in different tumor cell populations, the potential of combining this strategy with established therapies and the broad applicability of this approach to multiple cancer types highlight the significance of these findings.

## 1. Introduction

Glioblastoma (GBM) is the most aggressive primary brain tumor in adults. This tumor is highly heterogenous and is characterized by increased proliferation, angiogenesis, infiltration into the brain parenchyma and resistance to all current treatment modalities [1,2,3]. GBM remains an incurable disease and the median survival of GBM patients has remained around 14–16 months over the past several decades [4]. The 2021 WHO classifies adult diffuse glioma into three categories: low grade glioma (LGG, isocitrate dehydrogenase (IDH) mutant), oligodendroglioma (IDH-mutant and 1p/19q-codeleted) and glioblastoma (IDH-wild type, WHO grade 4, referred to here as GBM) [5]. These subtypes exhibit distinct genetic, molecular signatures, and cellular phenotypes [6]. GBM is also often categorized into different molecular subtypes (classical, proneural, neural and mesenchymal) based on genetic and molecular characteristics [7]. Current standard-of-care-treatment for GBM includes surgery followed by local radiation and treatment with temozolomide (TMZ) [8].

GBM contains a small subpopulation of cancer stem cells [9,10], (i.e., glioma stem cells [GSCs]) that are characterized by self-renewal capability, multi-lineage differentiation potential, and the ability to generate xenografts that recapitulate the paternal tumors in nude mice. GSCs have been implicated in tumor infiltration, treatment resistance and tumor recurrence [11,12,13]. Therefore, developing novel therapies targeting these cells is of great importance for the treatment of GBM and improving patient prognosis. The molecular mechanisms by which GSCs contribute to the different GBM subtypes, therapy resistance and tumor recurrence are very complex and just beginning to be understood. Therefore, the development of new therapies targeting GSCs is challenging and represents an urgent and unmet need. An emerging group of proteins found to be involved in different types of cancer, including GBM, is the TMED family.

TMED (Trans-Membrane Emp24 protein transport Domain, p24) proteins are a family of type-I transmembrane proteins that facilitate bi-directional transport at the ER-Golgi interface [14,15,16,17,18]. In vertebrates, the TMED family consists of ten cargo receptors, which physically link cargo with vesicle coat subunits to efficiently and selectively recruit proteins into emerging COP vesicles [14]. Specifically, evidence indicates that TMED proteins modulate the transport of diverse groups of cargo [15,19], regulating both forward and retrograde trafficking. As a consequence of their role in nascent protein trafficking, the TMED members are involved in embryonic development and contribute to various physiological and pathological conditions [20,21].

Recent studies have shown a significant relationship between multiple TMED members and progression, aggressiveness and metastasis in different types of cancers [20,21]. Bioinformatic analyses and experimental data have demonstrated an association of different TMED members (including TMEDs 2,3,5,7,9, and 10) with tumor progression and aggressiveness, and that the upregulation of different TMED members in cancer is associated with poor prognosis [20,22,23]. Several molecular mechanisms were suggested to mediate TMEDs’ effect on cancer, including the TGF-ɑ [24,25], WNT [24,26,27] and PI3K/AKT signaling pathway [28].

Interestingly, TMED family members were also found to play a role in GBM progression and aggressiveness [29,30]. An in-depth study investigating this relationship demonstrated a novel role for TMED2 in EGFR signaling [30], a process which has been shown to be crucial for promoting glioma development [31]. The study showed that TMED2 is involved in the recycling of EGFR upon EGF stimulation, thereby increasing EGFR stability and promoting malignant behavior [30]. In addition to TMED2, a recent bioinformatics study demonstrated a positive correlation between the expression of TMED4 and TMED9 and glioma aggressiveness and a negative association with patient prognosis [32]. The expression and role of TMED proteins in general and TMED9 in particular has not yet been reported in the GSC population.

Importantly, our previous work, focusing on misfolding diseases, has revealed BRD4780, a small molecule compound that targets TMED9 and promotes its delivery into lysosomes, where it is degraded and removed from the cell [33,34]. In view of the potential role of TMED9 as an oncogene in GBM, and the identification of a TMED9-targeting compound, we aimed to (i) study the expression and function of TMED9 in GSCs and (ii) investigate the effects of pharmacologically targeting TMED9 using BRD4780 as a potential anti-tumor treatment.

## 2. Materials and Methods

### 2.1. GSC and Neural Cell Cultures

Glioblastoma patient-derived cancer stem cell cultures were obtained from the live biobank collection at the Hermelin Brain Tumor Center, Henry Ford Hospital (Detroit, MI, USA). GSCs were isolated from fresh GBM samples and characterized as previously reported [35,36]. The GSCs were maintained in neurosphere medium (DMEM-F12 1:1, glutamine 10 mM, HEPES buffer 10 mM, and sodium bicarbonate 0.025%) supplemented with basic fibroblast growth factor and epidermal growth factor (20 ng/mL each). The GSCs displayed self-renewal ability, expressed key stem cell markers (OCT4, nestin, and CD44) and successfully generated glioma xenografts when transplanted into nude mice [37]. Comprehensive details of the GSCs used in this study can be found in Table S1.

Differentiated cancer cells (DGSCs) were generated by culturing GSCs in DMEM supplemented with 5% FBS for three days. This process led to the loss of spheroid-forming capacity and a significant reduction in Sox2 and OCT4 expression.

Human microglia and primary human astrocytes immortalized with hTERT were sourced from Applied Biological Materials (Richmond, BC, Canada). Human neurons were obtained from ScienCell (Carlsbad, CA, USA). Cells were maintained in growth media and conditions specified by the manufacturer [38]. All GSCs and neural cell cultures were regularly screened for mycoplasma contamination using a mycoplasma PCR detection kit, with no contamination detected.

### 2.2. DIPG Cultures

Patient-derived DIPG cultures [39] were cultured as spheroids in neurosphere medium (DMEM-F12 1/1, L-glutamine 10 mM, HEPES buffer 10 mM and sodium bicarbonate 0.025%) supplemented with 20 ng/mL basic fibroblast growth factor (bFGF) and epidermal growth factor (EGF).

### 2.3. Real-Time PCR

Total RNA was isolated from GSC, astrocyte, neural stem cells (NSC), neuron and microglia cultures using the RNeasy mini kit, according to the manufacturer’s protocol (Qiagen, Frederick, MD, USA). Reverse transcription was performed using 2-μg total RNA, as previously described. Reactions were carried out on an ABI VIIA7 Sequence Detection System (Applied Biosystems, Foster City, CA, USA), and cycle threshold (Ct) values were generated using Bio-Rad CFX Maestro 2.0. GAPDH RNA levels served as internal controls.

### 2.4. TMED9 Silencing

To knock down TMED9 expression in GSCs, cells were transfected with small interfering RNAs (siRNA) targeting TMED9. The TMED9 siRNA used was obtained from Santa Cruz Biotechnology, Dallas, TX, USA (Cat# sc-78430), and a non-targeting siRNA served as a negative control. Transfections were performed according to the manufacturer’s instructions. siRNA and transfection reagent were diluted separately in neurosphere medium, mixed gently, and incubated for 5 min at room temperature. The siRNA-reagent complexes were added dropwise to cells and incubated for 4–6 h. After this incubation period, fresh medium was added to the cells. Cells were cultured for 72 h before subsequent analyses. Western blotting confirmed the knockdown efficiency.

### 2.5. Western Blot and SDS-PAGE Gel Electrophoresis

Cells were harvested and lysed on ice using M-PER lysis buffer (Thermo Fisher, Waltham, MA, USA,#78501) supplemented with a protease inhibitor cocktail (1:100 dilution) (Sigma, Burlington, MA, USA, #P8340) and, if required, a phosphatase inhibitor (1:100 dilution). Lysates were collected, vortexed briefly every 5 min, and stored at −20 °C. Protein concentrates were determined using the Lowry Protein Assay. Protein lysates were normalized based on concentration and mixed with 4× sample buffer containing beta mercaptoethanol. Samples were then heated to 100 °C for 5 min prior to loading onto gel.

Proteins were separated by SDS-PAGE on polyacrylamide gels at 120 V, in tris-glycine buffer, and then transferred to PVDF membrane. Membranes were blocked in quick blocking solution (Biorad, Hercules, CA, USA, #12010020) for 5 min and then incubated with primary antibody for 1 h at room temperature. Following three washes with PBS-T, membranes were incubated with secondary antibodies, then washed three times with PBS-T and incubated with ECL (Millipore, Burlington, MA, USA, #WBLUF0500) and immunoreactive bands were imaged.

### 2.6. Transwell Migration Assay

Cell migration was assessed using transwell chambers equipped with 8 μm pore filters (BD Biosciences, San Jose, CA, USA). GSCs were seeded in transwell chambers at a density of 20,000 cells per well, and the chambers were incubated for 18–20 h in culture medium containing 10% FBS in the bottom chambers. Following incubation, non-migratory cells on the upper membrane surface were removed, while the cells that had migrated to the lower surface were stained with 0.02% crystal violet for 20 min. The stained cells were then examined under a microscope, and the number of migrating cells was quantified.

### 2.7. Neurosphere Formation (Self-Renewal) Assay

The ability of GSCs to form secondary neurospheres was analyzed as recently reported [27,30]. Cells were plated in densities of 100 cells/well through limiting dilution and the number of neurospheres/well was determined following 12 days for five different wells. Spheres that contained more than 20 cells were scored. The results are presented as percentages of maximal neurospheres formed in treated compared to control cells.

### 2.8. Cell Viability and Cell Death

To quantify cell death in GSCs, cells were plated in 96-well Phenoplates. DRAQ7 was added to the medium at a ratio of 1:5000 to analyze cell death. Treatments were added directly to medium upon plating of cells, and plates were imaged immediately after plating and every 24 h, using the Opera Phenix high-content screening system.

### 2.9. Image Analysis

For live cell image analysis, cells were identified by Digital Phase Contrast. DRAQ7-positive dead cells were then analyzed by Cy5 intensities above a determined threshold. The percentage of dead cells (dead cells/all cells) × 100 was calculated for each treatment at each given time point.

### 2.10. Propidium Iodide Uptake

Propidium iodide (PI) staining was performed to evaluate cell death in differentiated glioblastoma stem cells (DGSCs). GSCs were differentiated as described above. After the experimental treatments, cells were dissociated into single cells using trypsin-EDTA and washed twice with phosphate-buffered saline (PBS). The cell suspension was centrifuged at 300× *g* for 5 min, and the pellet was resuspended in 300 µL of PBS. PI (Sigma-Aldrich, #P4170) was added to the cell suspension at a final concentration of 2 µg/mL and incubated at room temperature for 30 min in the dark. Stained cells were analyzed using a flow cytometer (Fortessa) with excitation at 488 nm and emission at 617 nm. A minimum of 10,000 events per sample were recorded. Data was analyzed using FlowJo.

### 2.11. LDH Assay

Cell death was also evaluated using the LDH cytotoxicity detection kit (Roche #11644793001), which quantifies lactate dehydrogenase (LDH) levels in culture supernatants according to the manufacturer’s instructions. Growth medium was centrifuged, mixed with prepared solution, and incubated for 30 min. After 30 min, absorbance was read using an ELISA reader at 490 nm.

### 2.12. TCGA Data Analysis

Expression data from GBM and LGG patients, along with healthy tissue samples from GTEx, were downloaded from the XENA data portal (cohort: “TCGA TARGET GTEx”, dataset: gene expression RNAseq—RSEM norm_count, version: 2018-08-08). These data, obtained from the GDC Data Portal and GTEx, were normalized together. Clinical data were sourced from both XENA and a study on the molecular profiling of glioma subtypes.

TMED9 expression levels were evaluated in LGG and GBM and analyzed in relation to patient survival. Expression data are displayed graphically, with median values and interquartile ranges noted. Mean expression differences between groups were assessed using one-way ANOVA, followed by Tukey’s corrected two-sample tests, which adjust for multiple comparisons and preserve the family-wise error rate. Kaplan-Meier analysis was employed to estimate survival time from diagnosis to death or last follow-up. Survival differences between groups were determined using the log-rank test, while Cox regression modeling was applied to develop multivariable survival modes, including mRNA expression, age at diagnosis, IDH mutation status, and tumor grade. Graphs were generated using R (v.4.4.1).

### 2.13. Statistical Analysis

All experiments were conducted a minimum of three times. Statistical significance was assessed using a two-tailed unpaired Student’s *t*-test for the comparison of two groups, while-way ANOVA followed by Tukey’s post-hoc test was applied for analyses involving more than two groups. A two-way ANOVA was performed to evaluate interactions between two treatments. Unless otherwise specified, error bars indicate mean ± SEM unless otherwise stated. Statistical significance is denoted as follows: * *p* < 0.05, ** *p* < 0.0021, *** *p* < 0.0002, **** *p* < 0.0001.

## 3. Results

### 3.1. High TMED9 Expression Is Associated with Aggressive Tumors and Poor Prognosis

**Figure 1:**
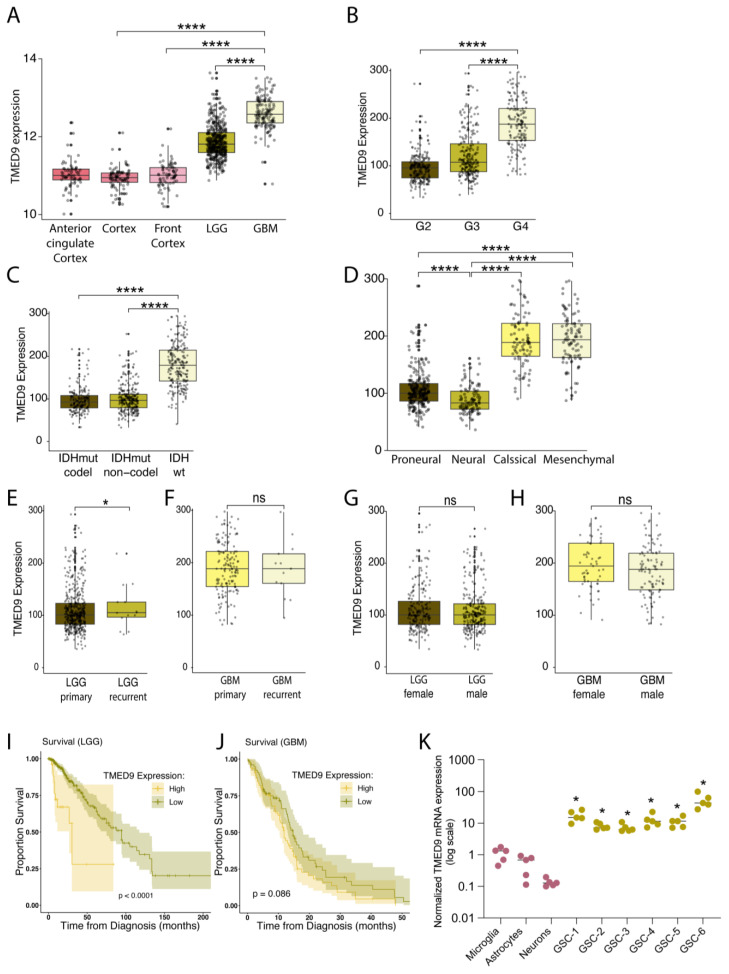
TMED9 is upregulated in GBM and GSCs and is associated with poor survival of patients. (**A**) TMED9 RNA expression is upregulated in low-grade glioma (LGG, G2, *n* = 220; G3, *n* = 240) patients, with a further increase observed in glioblastoma (GBM, G4, *n* = 140) patients. Data from glial tumors was determined according to The Cancer Genome Atlas, TCGA data portal and compared to healthy brain tissues (Anterior cingulate Cortex; *n* = 69, Cortex; *n* = 92, and Front Cortex; *n* = 78), Wilcoxon *t*-test. (**B**) Grade 4 gliomas exhibit significantly higher TMED9 expression compared to grade 2 and grade 3 gliomas. Data was processed as in (**A**). (**C**) TMED9 expression in glioma tumors (GBM and LGG) is significantly higher in IDH wild-type (IDHwt) tumors compared to IDH mutant (IDHmut) tumors. IDHwt: *n* = 229, IDHmut-non-codel: *n* = 268, IDHmut-codel: *n* = 171, Wilcoxon *t*-test. (**D**) Mesenchymal GBM subtypes exhibit significantly elevated TMED9 expression compared to proneural and neural subtypes. Proneural, *n* = 250; Neural, *n* = 109; Classical, *n* = 87; Mesenchymal, *n* = 100, Wilcoxon *t*-test. (**E**) Recurrent LGG tumors (*n* = 14) show significantly higher TMED9 expression compared to primary LGG tumors (*n* = 446). (**F**) No significant difference in TMED9 expression between primary (*n* = 147) and recurrent (*n* = 13) GBM tumors. (**G**,**H**) No significant gender differences in TMED9 expression between female (*n* = 204) and male (*n* = 256) with LGG tumors (**G**) or female (*n* = 57) and male (*n* = 103) with GBM tumors (**H**). (**I**) TMED9 upregulation is significantly associated with poor survival in LGG patients. Overall survival was determined using a Kaplan–Meier survival model, with the *p*-value calculated using the log-rank test. (**J**) TMED9 expression trends toward an association with poor survival in GBM patients but does not achieve statistical significance. Survival was determined as in (**I**). (**K**) RT-PCR analysis of TMED9 mRNA levels in six different patient-derived GSCs compared to normal astrocytes, microglia and neurons (*n* = 5, * *p* < 0.05, compared to neurons). * *p* < 0.05, **** *p* < 0.0001, ns, not significant.

To examine the potential role of TMED9 in GBM and provide support for the therapeutic applicability of the small molecule BRD4780, we analyzed available databases and investigated TMED9 expression in various brain tissues and astrocytic tumors. Utilizing sample data from TCGA and GTEx databases, we first tested the expression of TMED9 in normal brain tissues compared to tissues derived from LGG and GBM tumors. Our analysis indicated that TMED9 expression is upregulated in samples derived from LGG patients, and to a larger degree in GBM patients (Figure 1A). Similarly, elevated TMED9 levels were observed in grade 4 gliomas compared to grade 2 or 3 gliomas (Figure 1B). Interestingly, we found that TMED9 expression was significantly upregulated in IDHwt when compared with IDHmutant tumors (Figure 1C) and in mesenchymal compared with proneural and neural subtypes (Figure 1D), indicating that aggressive tumors with poor prognosis are associated with elevated levels of TMED9. Our analysis of TMED9 expression in primary and recurrent glioma tumors demonstrated a significant increase in TMED9 in recurrent LGG tumors (Figure 1E) with no significant difference in recurrent GBM tumors (Figure 1F). Notably, no difference in TMED9 expression was found between males and females in either LGG (Figure 1G) or GBM tumors (Figure 1H). Finally, our analysis shows that TMED9 upregulation is significantly associated with poor survival in LGG patients (*p* < 0.001, Figure 1I), providing additional support for the comprehensive findings previously reported by Ullah et al. [32] Notably, while TMED9 expression was elevated in GBM, its association with poor survival was close to but did not achieve statistical significance (*p* = 0.086, Figure 1J).

The emerging findings associating TMED9 with GBM development and aggressiveness motivated us to study the potential role of TMED9 in the glioma stem cell population. GSCs are of great importance to cancer progression and resistance to treatment, yet the functional involvement of TMED9 in the GSC population remains undetermined. We therefore tested TMED9 expression level in GSCs derived from six different IDHwt tumor specimens (for patient information see Table S1) and compared them to normal brain cells. Importantly, TMED9 levels were significantly higher in the patient-derived GSCs as compared to normal microglia, astrocytes, and neurons (Figure 1K). The documented correlation between TMED9 and GBM aggressiveness, coupled with the elevated TMED9 expression levels in these difficult-to-treat GSCs, prompted us to study the function and therapeutic potential of TMED9 in the GSC subpopulation.

### 3.2. Genetic Removal of TMED9 Inhibits Self-Renewal and Migration of Patient-Derived GSCs

**Figure 2:**
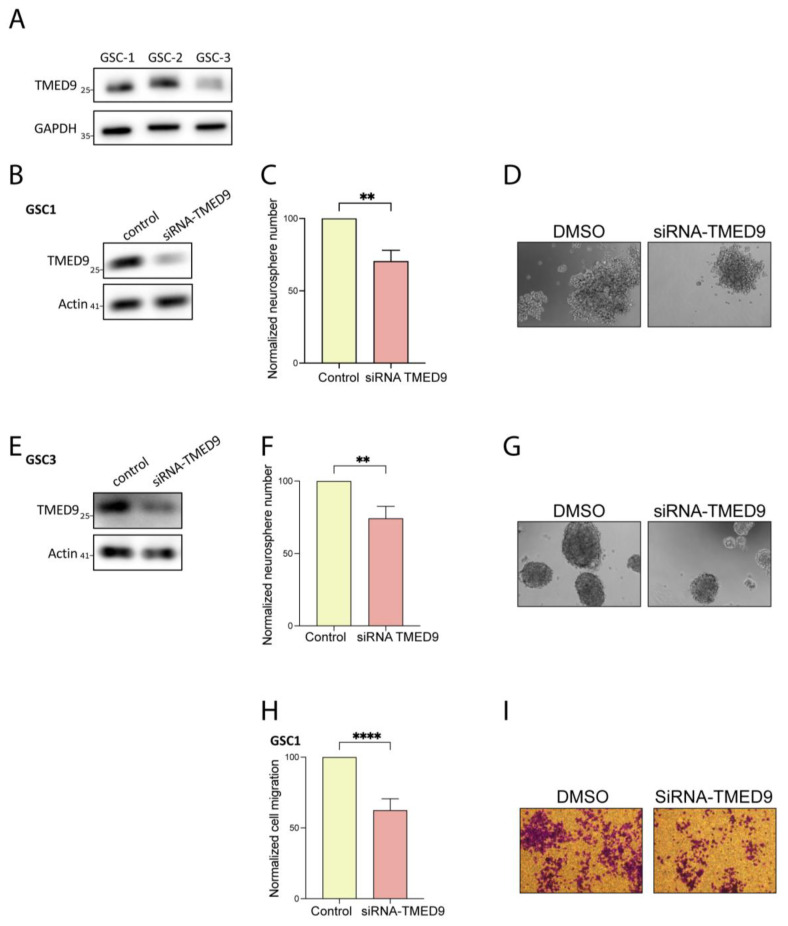
TMED9 silencing inhibits self-renewal and migration of GSCs. (**A**) Western blot analysis of TMED9 levels across different patient-derived GSCs, confirming TMED9 expression (*n* = 3). (**B**,**E**) Partial knockdown of TMED9 in GSCs using siRNA, demonstrated by Western blot analysis (*n* = 3). (**C**,**D**) Quantification (**C**) and representative images (**D**) of secondary neurosphere formation following TMED9 silencing in GSC1. For self-renewal analysis, GSCs were plated at 100 cells/well in 96-well plates, and the number of neurospheres per well was quantified after 14 days. Self-renewal is determined as % of normalized control cells (*n* = 3). (**F**,**G**) Quantification (**F**) and representative images (**G**) of secondary neurospheres formation following TMED9 silencing in GSC3 (*n* = 3). (**H**,**I**) Quantification (**H**) and representative images (**I**) of cell migration in control and TMED9 silencing GSCs. Cell migration was analyzed using a transwell migration assay. Experiments were performed in triplicates and repeated three times. Five fields were analyzed for each well (*n* = 3). ** *p* < 0.0021, **** *p* < 0.0001.

First, we investigated whether the TMED9 protein is expressed in the GSC population. Through our examination of three different patient-derived GSCs, we confirmed the presence and abundance of TMED9 protein in this critical cell population (Figure 2A). To delineate the potential role of TMED9 in different tumorigenic functions of GSCs, we silenced TMED9 expression using targeted siRNAs. The application of siRNA to two different GSCs resulted in a partial removal of TMED9 (Figure 2B,E). Notably, GSCs possess the ability for self-renewal, thus maintaining cancerous properties and preventing effective treatment. We therefore asked whether TMED9 silencing would affect this essential function of GSCs. Although the decrease in TMED9 abundance was only partial, it resulted in a significant inhibition (20–25%) of self-renewal, as determined by measuring the generation of secondary neurospheres (Figure 2C,D,F,G). Importantly, in addition to their self-renewal capacity, GSCs exhibit a strong migratory ability, a function that is closely linked to the mesenchymal phenotype, which facilitates cell invasion and promotes disease progression. A decrease in TMED9 abundance successfully inhibited the migration of GSCs by 30% (Figure 2H,I). This is the first time, to the best of our knowledge, that TMED9 has been shown to mediate cancer stem cell tumorigenesis.

### 3.3. BRD4780 Targets and Removes TMED9 from Patient-Derived GSCs and Induces Anti-Tumor Effects

**Figure 3:**
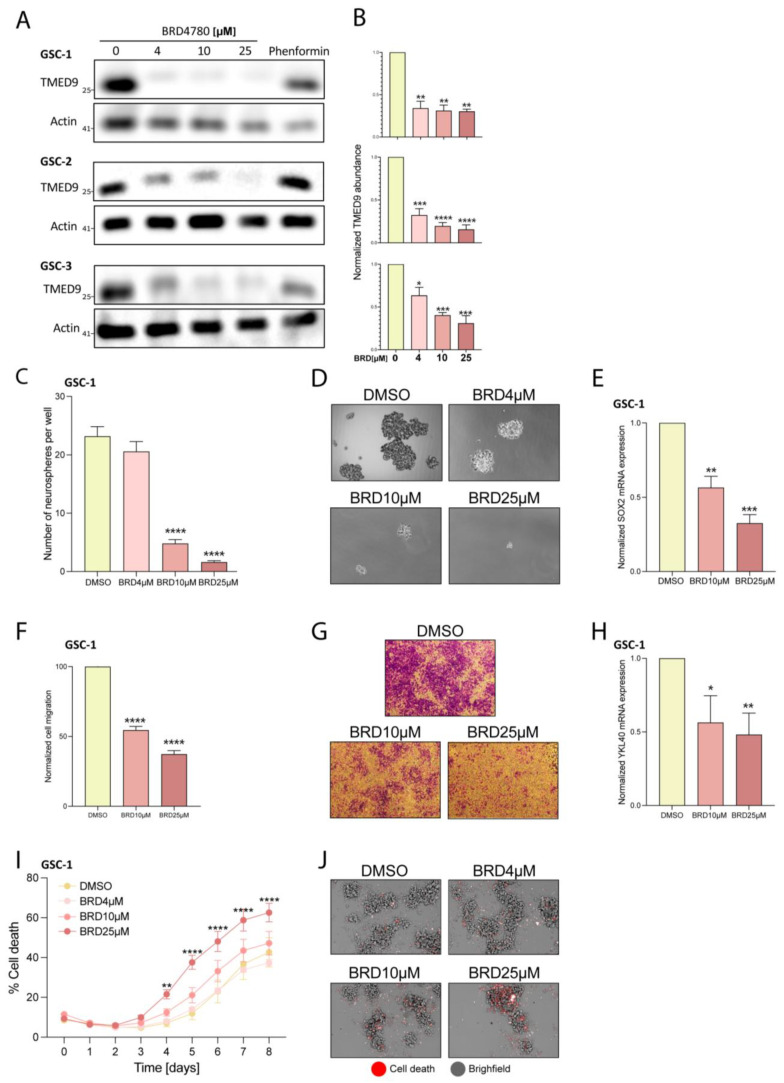
BRD4780 targets TMED9 and inhibits GSC tumorigenic functions. (**A**,**B**) Western blot (**A**) and quantification (**B**) of TMED9 levels in three different patient-derived GSCs treated with various concentrations of BRD4780 for 48 h (*n* = 3). (**C**,**D**) Quantification (**C**) and representative images (**D**) of neurospheres after 14 days of BRD4780 treatment (*n* = 3). (**E**) RT-PCR analysis of SOX2 mRNA levels in GSCs treated with BRD4780 for 48 h (*n* = 3). (**F**,**G**) Quantification (**F**) and representative images (**G**) of cell migration in GSCs treated with BRD4780 (*n* = 3). (**H**) RT-PCR analysis of YKL40 mRNA levels in GSCs treated with BRD4780 for 48 h (n = 3). (**I**,**J**) Quantification (**I**) and representative images (**J**) of BRD4780-induced cell death in GSCs over 8 days. Cell death was assessed using DRAQ7 dye (red) and calculated as the percentages of DRAQ7 positive cells. (*n* = 3). Separated channels are displayed in Figure S1K. * *p* < 0.05, ** *p* < 0.0021, *** *p* < 0.0002, **** *p* < 0.0001.

BRD4780 is a small molecule which targets and promotes the removal of TMED9 in normal epithelial cells [33,40,41]. In light of our findings on TMED9′s oncogenic function in patient-derived GSCs, we sought to apply BRD4780 and test its efficacy in clearing TMED9 from GSCs, and its potential inhibition of tumorigenic functions [33]. We first analyzed the drug’s effect on TMED9 expression in various GSCs. Following 48 h of treatment, BRD4780 decreased TMED9 expression in a dose-dependent manner in three different patient-derived GSCs (Figure 3A,B). Treatment of the cells with the biguanide phenformin, which was previously demonstrated by us to target GSCs [42] did not induce a significant change in TMED9 expression in these cells. We then studied the drug’s effect on formation of secondary neurospheres after 14 days of treatment. BRD4780 exerted a remarkable inhibitory effect on GSCs self-renewal, demonstrating almost complete inhibition of self-renewal at the highest concentration applied (Figure 3C,D). This effect was also demonstrated in two other patient-derived GSCs (Figure S1A,B,D,E). To explore the cellular mechanism associated with this inhibition, we tested the effect of BRD4780 on the mRNA levels of SOX2. SOX2 is a key stemness factor which cooperates with multiple co-factors to drive the transition of terminally differentiated somatic cells to induced pluripotent stem cells [43,44]. It has also been linked to stemness in various cancers including glioblastoma [45,46,47]. Following treatment, we observed a marked and dose dependent reduction in SOX2 expression (Figure 3E), demonstrating that BRD4780 reduces the stemness of GSCs. This effect was reproduced in two other patient-derived GSCs (Figure S1C,F). Next, we assessed the effect of TMED9 clearance on GSC migration. Treatment with BRD4780 dose-dependently reduced GSC migration (Figure 3F,G). This effect was consistently reproduced in two other patient-derived GSCs (Figure S1G–J). To further characterize this effect, we analyzed the expression of YKL-40, which is highly expressed in mesenchymal GBM and implicated in epithelial-mesenchymal transition and cell migration [48,49,50]. Interestingly, YKL40 expression was markedly reduced upon BRD4780 treatment (Figure 3H), further demonstrating the effect of BRD4780 as an inhibitor of glioma cell migration. Lastly, we studied the drug’s effect on cell viability over time, by live imaging using the membrane impermeable DRAQ7 dye as a readout for cell death. BRD4780 application resulted in a dose-dependent induction of cell death, at multiple points over the course of one week (Figure 3I,J and Figure S1K). Importantly, healthy brain cells (Figure S1L) and normal epithelial cell lines [33] subjected to BRD4780 under similar conditions do not undergo cell death or visible damage.

### 3.4. BRD4780 Enhances the Effect of TMZ

**Figure 4:**
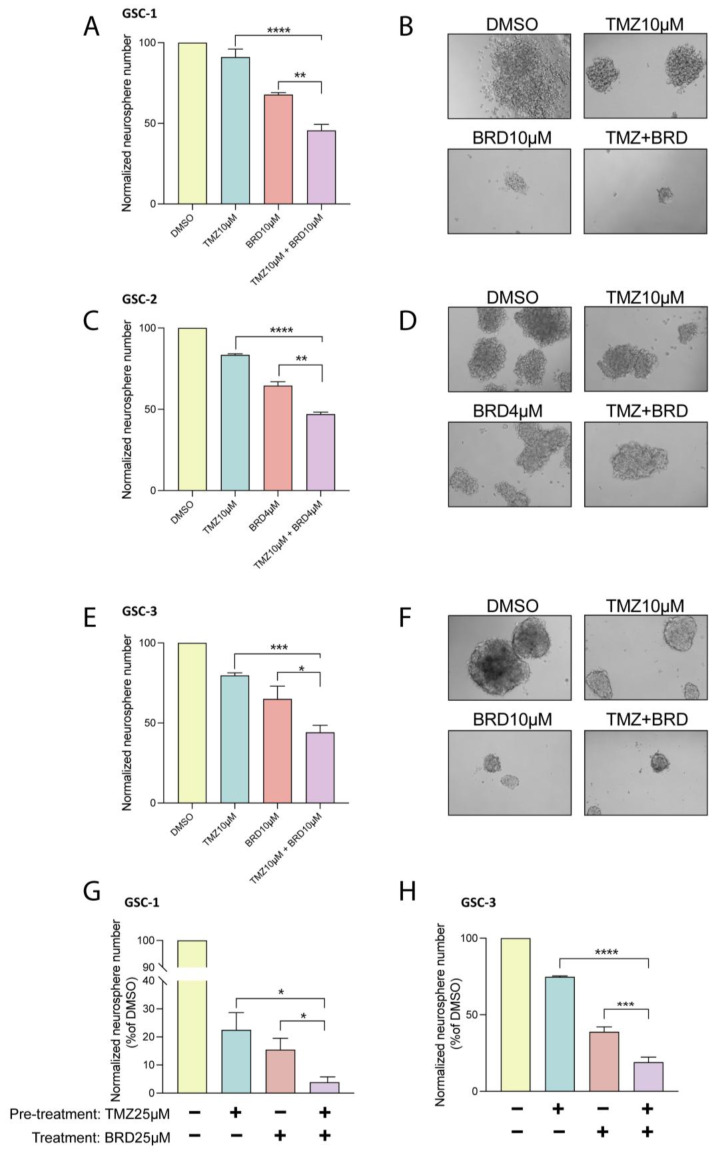
BRD4780 enhances the efficacy of temozolomide (TMZ). (**A**–**F**) Quantification (**A**,**C**,**E**) and representative images (**B**,**D**,**F**) of neurosphere formation in GSCs treated with TMZ (10 μM), BRD4780 (4 or 10 μM), or a combination of TMZ and BRD4780. Combined treatment significantly reduces self-renewal compared to single treatments (*n* = 3). (**G**,**H**) Quantification of neurosphere formation following sequential treatment with TMZ (72-h pretreatment) and BRD4780, showing enhanced inhibition of self-renewal compared to individual treatments. * *p* < 0.05, ** *p* < 0.0021, *** *p* < 0.0002, **** *p* < 0.0001.

TMZ is a standard treatment for GBM patients following surgery. We therefore examined how BRD4780 affects GSCs’ response to TMZ. We found that the combined treatment with BRD4780 and TMZ (25 μM) significantly decreased self-renewal of three different patient-derived GSCs when compared to each treatment alone (Figure 4A–F). Moreover, to test for the potential benefit of sequential application of the two treatments, we assessed the effect of pretreating GSCs with TMZ for 72 h, followed by BRD4780 treatment. The combined treatment demonstrated a markedly stronger inhibition of self-renewal compared to each individual treatment (Figure 4G,H). We therefore suggest that combining BRD4780 with TMZ could yield a more effective outcome in GBM patients.

### 3.5. BRD4780 Targets and Removes TMED9 from Differentiated Tumor Cell Progeny (DGSCs) and Promotes Anti-Tumor Effects

**Figure 5:**
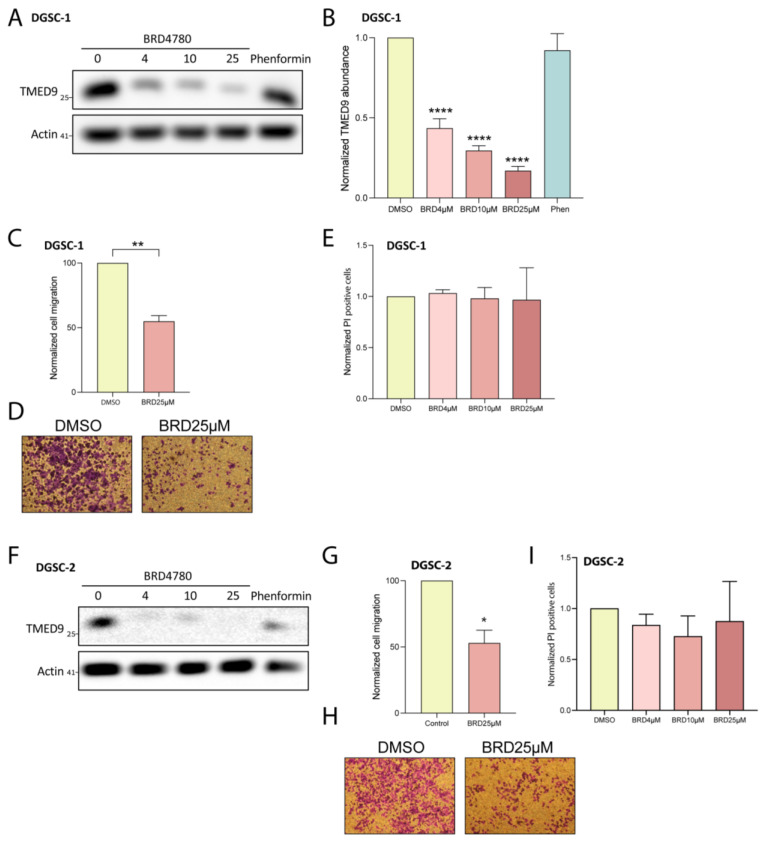
BRD4780 inhibits TMED9 and reduces tumorigenic functions in differentiated GSCs (DGSCs). (**A**,**B**) Western blot (**A**) and quantification (**B**) showing dose-dependent reduction of TMED9 levels in DGSC-1 treated with BRD4780 (*n* = 3). (**C**,**D**) Quantification (**C**) and representative images (**D**) of DGSC-1 migration, demonstrating significant inhibition following treatment with 25 μM BRD4780 (*n* = 3). (**E**) Propidium iodide assay for cell death in DGSC-1 treated with BRD4780. No significant cell death is observed, indicating that BRD4780 reduces migration without inducing cytotoxicity (*n* = 3). (**F**) Western blot showing TMED9 reduction in DGSC-2 treated with BRD4780 (*n* = 3). (**G**,**H**) Quantification (**G**) and representative images (**H**) of cell migration in DGSC-2 treated with BRD4780 (*n* = 3). (**I**) Propidium iodide assay for cell death in DGSC-2 treated with BRD4780. No significant cell death is observed, indicating that BRD4780 reduces migration without inducing cytotoxicity (*n* = 3). * *p* < 0.05, ** *p* < 0.0021, **** *p* < 0.0001.

GSCs constitute a small percentage of the cellular makeup of GBM, with the majority of the tumor composed of differentiated cells. We therefore examined the anti-tumorigenic effects of BRD4780 on differentiated tumor cell progeny derived from GSC1 and GSC2 (differentiated GSC; DGSCs, Figure S2A). The application of BRD4780 dose-dependently reduced the abundance of TMED9 in two DGSCs (Figure 5A,B,F). We then tested the drug’s effect on migration of differentiated tumor cells. BRD4780 inhibited the migration of differentiated tumor cells by nearly 50% (Figure 5C,D,G,H). Notably, BRD4780 did not alter propidium iodide uptake in DGSCs, indicating that cell death was not induced (Figure 5E,I). This result suggests that TMED9 may be differentially involved in supporting tumorigenic functions in differentiated tumor cells versus in the stem cell population as the latter did demonstrate significant cell death in response to TMED9 inhibition (Figure 3I,J). Altogether, these findings point to the oncogenic role of TMED9 not only in the stem cell population, but also in the differentiated tumor cell progeny.

### 3.6. BRD4780 Drives Its Anti-Tumor Effects Through the Lysosomal Removal of TMED9

**Figure 6:**
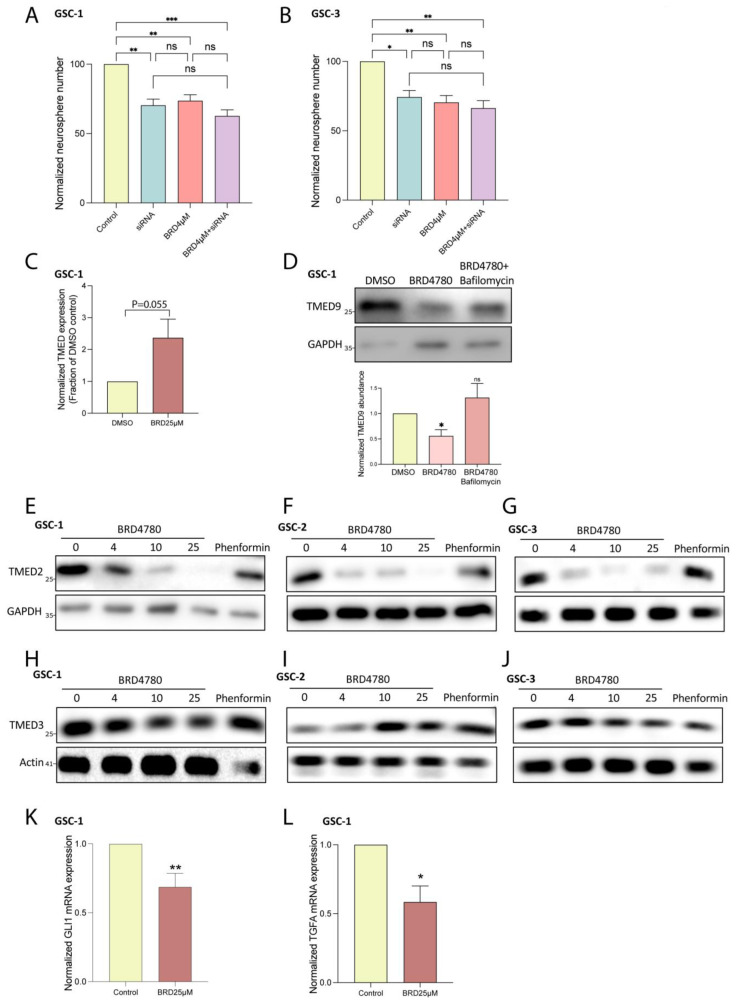
BRD4780 exerts anti-tumor effects by TMED9 removal. (**A**,**B**) Quantification of neurosphere formation in GSCs treated with siRNA targeting TMED9, BRD4780, or a combination of siRNA and BRD4780. Combined treatment shows no additive effect, indicating BRD4780′s primary mechanism is TMED9 removal (*n* = 3). (**C**) RT-PCR analysis showing that TMED9 mRNA levels are not reduced by BRD4780, suggesting degradation rather than transcriptional regulation (*n* = 3). (**D**) Bafilomycin A prevents BRD4780-induced TMED9 degradation, confirming a lysosomal degradation mechanism. All treatments were applied for 24 h followed by protein extraction and western blot analysis (*n* = 4). (**E**–**G**) Western blot analysis of TMED2 in different GSCs treated for 48 h with different concentrations of BRD4780 (*n* = 3). (**H**–**J**) Western blot analysis of TMED3 in different GSCs treated for 48 h with different concentrations of BRD4780 (*n* = 3). (**K**,**L**) RT-PCR analysis showing that *GLI1* (**K**) and TFGA (**L**) mRNA levels are reduced by BRD4780 (*n* = 3). * *p* < 0.05, ** *p* < 0.0021, *** *p* < 0.0002, ns, not significant.

To gain insight into BRD4780′s mechanism of action, we first investigated whether the drug exerts its anti-tumor effects by altering TMED9 abundance. When testing BRD4780′s effect on GSC self-renewal, the application of low dose of BRD4780 (4 μM) resulted in similar inhibition to that of TMED9 silencing (Figure 6A,B, blue vs. pink bars). Under those conditions, BRD4780 treatment in combination with TMED9 silencing had no added effect (Figure 6A,B, purple vs. blue and pink bars). These findings suggest that BRD4780′s anti-tumor effects are mainly driven by TMED9 removal. Next, we explored the mechanism by which BRD4780 mediates TMED9 removal. We first tested the drug’s effect on TMED9 mRNA expression. Not only did TMED9 expression not decrease, but rather, it demonstrated a slight increase, (Figure 6C) indicating that BRD4780 does not achieve its reduction of TMED9 via transcriptional regulation. Next, we tested whether the decreased abundance is driven by reduced protein stability and enhanced degradation. To this end, we applied bafilomycin A to inhibit lysosomal degradation and demonstrated that it stabilized TMED9 abundance, abolishing the removal induced by BRD4780 (Figure 6D). This indicates that BRD4780 removes TMED9 by promoting its lysosomal degradation.

TMED9 belongs to a family of TMED cargo receptors which can function either as monomers or within dynamic complexes, where the stability of one member can impact others [51,52,53]. As noted, additional TMED members were also shown to be associated with different types of cancers. Importantly, TMED2 was demonstrated to promote glioma tumorigenesis by mediating EGFR recycling transport. Genetic ablation of TMED2 was previously shown to induce the degradation of EGFR, therefore resulting in an anti-tumor effect. Given the oncogenic effect of TMED2 in glioma, we studied the potential effect of BRD4780 on TMED2 stability. BRD4780 significantly reduced the abundance of TMED2. This effect was observed in three different patient-derived GSCs (Figure 6E–G).

As mentioned, TMED family members are involved in tumor metastases across various cancers. Studies on colon cancer have highlighted the involvement of TMED9 in driving a pro-metastatic state in colon cancer models. In contrast to TMED9, the cargo receptor TMED3 was shown to have anti-metastatic effects [24]. These antagonistic functions are mediated by two opposing signaling loops activated by the different TMED members. While TMED9 upregulation led to the activation of a pro metastatic signaling pathway which includes *GLI1*, *CNIH4* and *TGFA,* the upregulation of TMED3 induced the anti-metastatic *WNT*-*TCF* pathway instead [24].

Given that TMED3 was shown to have an anti-metastatic effect, in contrast to TMED2/9, we tested the effect of BRD4780 on this cargo receptor. Interestingly, while BRD4780 removed TMED9 (Figure 3A,B) and TMED2 (Figure 6E–G) it had no significant effect on TMED3 abundance (Figure 6H). These results were reproducible in three different patient-derived GSCs (Figure 6H–J). Collectively, these results further support the antitumor effect of BRD4780, by preserving the anti-metastatic member TMED3 and reducing expression of oncogenic members TMED2 and TMED9 and their regulatory partners, *GLI1* and *TFGA*. Indeed, treatment of GSCs with BRD4780 reduced both *GLI1* (Figure 6K) and *TGFA* (Figure 6L) expression.

### 3.7. BRD4780’s Anti-Tumor Effect Is Also Observed in Pediatric Brain Tumors

**Figure 7:**
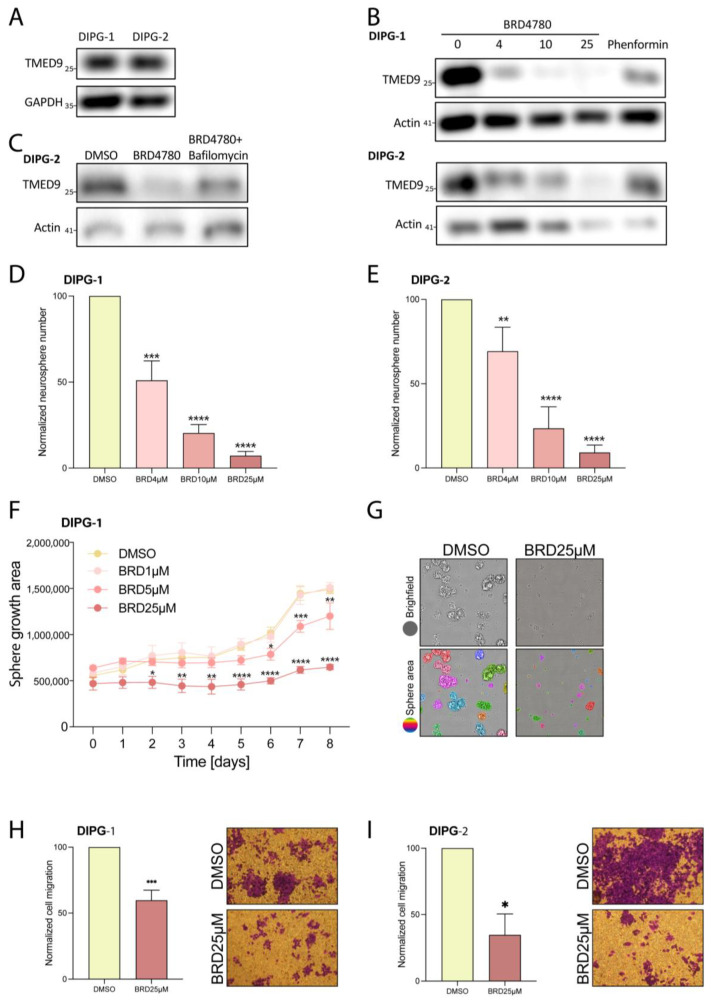
BRD4780 inhibits TMED9 and exerts anti-tumor effects in DIPG cells. (**A**) Western blot showing TMED9 abundance in two different DIPG cell lines. (**B**) Western blot analysis of TMED9 abundance following 48 h treatment of DIPG cells with different concentrations of BRD4780 (*n* = 3). (**C**) Bafilomycin A prevents BRD4780-induced TMED9 degradation, confirming a lysosomal degradation mechanism. All treatments were applied for 24 hrs followed by protein extraction and western blot analysis (*n* = 3). (**D**,**E**) Quantification (**D**) and representative images (**E**) of neurosphere formation in DIPG cells treated with BRD4780. All tested concentrations significantly reduce neurosphere formation. (**F**,**G**) Quantification (**F**) and representative images (**G**) of spheroid size reduction in BRD4780-treated DIPG cells for 8 days. Spheroid size was determined using image analysis by first identifying individual spheroids (as marked by different colors) and calculating their mean area. Images are displayed only for control and 25 μM BRD4780 after 6 day treatment. (**H**,**I**) Quantification (**H**) and representative images (**I**) of DIPG cell migration, showing significant inhibition following BRD4780 treatment. * *p* < 0.05, ** *p* < 0.0021, *** *p* < 0.0002, **** *p* < 0.0001.

To evaluate the broader applicability of BRD4780′s anti-tumor effects, we tested the drug on an additional type of glioma tumor. Diffuse intrinsic pontine glioma (DIPG), a subset of diffuse midline glioma (DMG), is a highly aggressive brain tumor that develops in the brainstem, primarily affecting children aged 5 to 10 years. This devastating condition is associated with poor prognosis, with a median survival of just 11 months [54]. A main driver of the disease is histone H3 K27 substitution of lysine to methionine that leads to significant dysfunction of epigenetic programming and signaling [55]. To test the applicability of BRD4780 to DIPG, we first demonstrated the presence of TMED9 in two different patient-derived DIPG stem cells (Figure 7A). We then applied BRD4780 and observed a dose-dependent removal of TMED9 in the two tested DIPG stem cells (Figure 7B). Similarly to GSCs, the reduction in TMED9 was achieved through its delivery to the lysosome and subsequent degradation (Figure 7C). Next, we tested the drug’s effect on self-renewal of DIPG cells and observed significant inhibition at all tested concentrations. Notably, the highest dose of the drug almost completely abolished neurosphere formation (Figure 7D,E). Moreover, the growth of the spheres was significantly inhibited, as detected by live imaging analysis of the spheroid size (Figure 7F,G).

Additionally, we analyzed the effect of BRD4780 on the migration of DIPG cells and found that it exerted a significant inhibitory effect (Figure 7H,I). These findings demonstrate the importance of TMED9 not only in adult glioma but also in pediatric glial tumors and the broader applicability of BRD4780 as a potential anti-tumor therapy.

## 4. Discussion

Gliomas, particularly glioblastoma (GBM), remain among the most challenging malignancies to treat due to their aggressive nature and resistance to conventional therapies. Despite advancements in surgery, radiotherapy, and chemotherapy, glioma patients continue to face a dismal prognosis. Glioma stem cells (GSCs) are a subpopulation of tumor cells with self-renewal capacity and multi-lineage differentiation. They are a major contributing factor to treatment resistance and tumor recurrence, highlighting the urgent need for novel therapeutic strategies targeting this resilient cell population.

Our study demonstrates that targeting TMED9, either by genetic perturbation or with the small molecule BRD4780, impairs key tumorigenic properties of GSCs, including self-renewal and migration. Additionally, we have shown that TMED9 removal exerts anti-tumorigenic effects in the differentiated tumor cell progeny. Importantly, this therapeutic approach is beneficial not only for adult gliomas but also for the highly aggressive pediatric DMG tumors. Our study therefore shows promise for broad applicability of TMED9 targeting across different glioma subtypes.

Given the growing body of studies linking various TMED proteins to multiple cancer types, we propose that targeting TMED9 could be broadened as a therapeutic strategy for other malignancies.

While our study highlights the impact of BRD4780 on both cancer stem cells and differentiated tumor cells, recent findings by Vredevoogd et al. emphasize the critical role of TMED inhibition in enhancing the sensitivity of immune cells to tumor cells [56]. Specifically, TMED9, among other TMED members, was shown to regulate the expression of programmed cell death protein 1 (PD-1) on CD8 T cells. Because PD-1 is an essential immune checkpoint contributing to T cell dysfunction, its suppression by TMED targeting has augmented T cell activity. Collectively, these results suggest that BRD4780 could also modulate immune responses, further amplifying its therapeutic potential.

A significant result of our study is the potential benefit of combining BRD4780 with TMZ, the current standard chemotherapy for GBM. Our findings demonstrate that this combined treatment resulted in an enhanced effect in reducing GSC self-renewal and migration, compared to each treatment alone. This enhanced effect could address one of the key challenges in GBM treatment, where tumors often exhibit resistance to TMZ. Therefore, combined TMED9 inhibition and TMZ treatment could represent a promising strategy to overcome resistance and achieve more durable responses in glioma patients.

From a mechanistic standpoint, our study builds on previous findings that TMED proteins play critical roles in cancer-related pathways. TMED2 has been shown to facilitate EGFR recycling, promoting receptor stability and tumor growth [30]. Thus, the removal of TMED2 results in EGFR degradation, reducing cancer cell proliferation. TMED9 itself has been implicated in the WNT/β-catenin signaling pathway [24,26,57,58], as well as in affecting growth factor signaling [26,29,59,60,61,62], both are key drivers of tumor progression and therapy resistance. Lastly, TMED9 was shown to mediate colon cancer metastases by activating the *GLI1*, *CNIH4* and *TGFA* regulatory pathway and opposing TMED3-WNT-TCF signaling [24]. Our results indicate that BRD4780 inhibited the expression of Gli1 and TGF⍺ in GSCs. In addition, BRD4780 decreased the expression of SOX2 and YKL40, which are involved in the stemness and mesenchymal transition of GSCs, respectively. Moreover, our work demonstrates that BRD4780 targets not only TMED9 but also the oncogenic TMED2 while it preserves the expression of anti-metastatic TMED3. By targeting TMED9, BRD4780 reduces the stemness and migration of the stem cell population while also disrupting pro-metastatic regulatory cascades. Collectively, these perturbations lead to the impairment of both stem cell maintenance and tumor cell survival.

An important question that remains unanswered is whether BRD4780 crosses the blood-brain barrier (BBB), a major hurdle in treating central nervous system tumors. Although the ability of BRD4780 to cross the BBB may be important for its potential application as a therapy for glioma patients, alternative administration modes can be employed such as intranasal administration [62], or encapsulation in nanoparticles [63,64], or exosomes [65] as was recently reported to other therapeutic agents. Our in vitro data strongly support the efficacy of BRD4780 in GSCs and DGSCs and its selective effects on tumor cells compared with human neural cells. In vivo studies will be important to fully validate the therapeutic potential of BRD4780 and TMED9 inhibition and would further contribute to our understanding of BRD4780′s pharmacokinetics, biodistribution, and ability to cross the BBB, as well as its impact on tumor growth, survival, and the immune response.

## 5. Conclusions

In conclusion, our findings demonstrate an essential oncogenic role for TMED9 in both adults and pediatric glial tumors. Importantly, this is the first time, to the best of our knowledge, that TMED9 is shown to play a major role in the function and tumorigenesis of brain tumor cancer stem cells and pediatric brain tumors. Our study identifies TMED9 targeting by silencing or the small molecule BRD4780 as a promising therapeutic approach to combat glioma aggressiveness and improve patient outcomes. The effectiveness of this approach for both stem cell and differentiated cell populations, the potential broad applicability of TMED9 targeting to various cancer types and the potential for combination with existing therapies underscore the significance of our findings. Future research should aim to address the in vivo effectiveness of TMED9 targeting and its effects on the tumor microenvironment to unlock its full potential as a novel cancer therapy.

## Data Availability

All data and materials reported in this paper will be shared by the lead contact upon request.

## Supplementary Materials

The following supporting information can be downloaded at: https://www.mdpi.com/article/10.3390/cells14110772/s1. Table S1: De-identified patient information of GSCs Figure S1: BRD4780 inhibits tumorigenic functions in additional GSC lines. (A,B,D,E) Quantification and representative images of secondary neurosphere formation in additional GSCs following BRD4780 treatment (*n* = 3). (C,F) RT-PCR analysis showing BRD4780 reduces SOX2 expression across GSC lines (*n* = 3). (G-J) Quantification and representative images of migration inhibition in BRD4780-treated GSCs (*n* = 3). (K) Images of separated channels of BRD4780’s dose-dependent induction of cell death (as calculated in Figure 3I). (L) Cell death was assessed in astrocytes, microglia, and neurons using an LDH assay (*n* = 3). Figure S2: Differentiated tumor cell progeny derived from GSCs. (A) Morphological differences between GSCs and DGSCs.
