# Supplementary material for: Targeting the Cargo Receptor TMED9 as a Therapeutic Strategy Against Brain Tumors
Source: Cells. 2025 May 23;14(11):772. doi: 10.3390/cells14110772 (PMC12153874; doi:10.3390/cells14110772)
Supplement: Supplementary file 1 [file cells-14-00772-s001.zip › cells-3521342-Supplementary Table1.pdf]

Supplementary Table 1. De-identified patient information of GSCs

Cell Line	Gender	MGMT	IDH status
**GSC-1**	M	U	WT
**GSC-2**	M	U	WT
**GSC-3**	F	U	WT
**GSC-4**	F	U	WT
**GSC-5**	F	M	WT
**GSC-6**	M	U	WT

For each GSC, the age, gender, MGMT (U-unmethylated and M-methylated), and IDH status are presented.
