# Supplementary figures and images for: Targeting the Cargo Receptor TMED9 as a Therapeutic Strategy Against Brain Tumors
Source: Cells. 2025 May 23;14(11):772. doi: 10.3390/cells14110772 (PMC12153874; doi:10.3390/cells14110772)

**Figure S1**

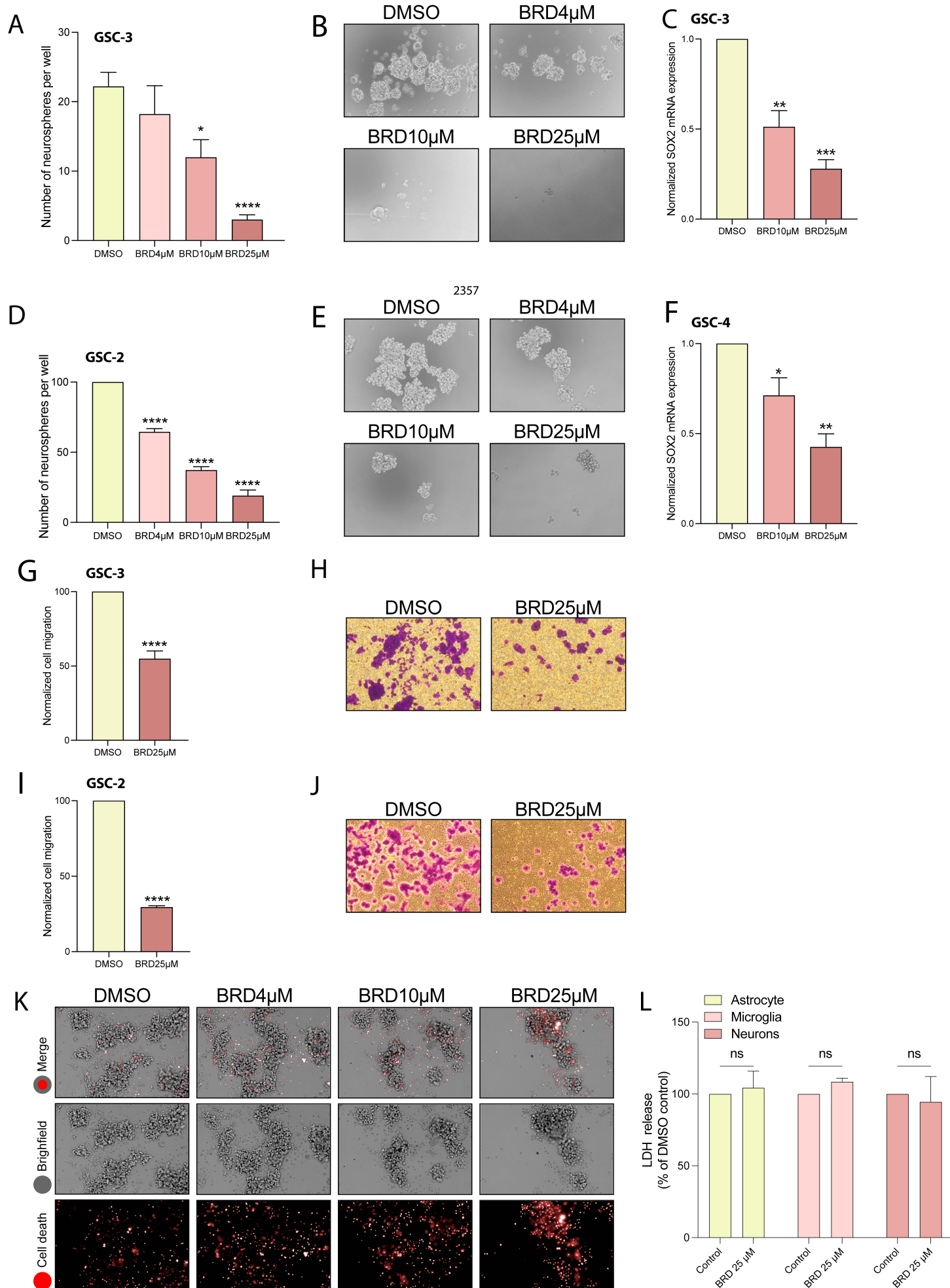

Supplement: Supplementary file 1 [file cells-14-00772-s001.zip › cells-3521342-FigureS1.pdf]

A

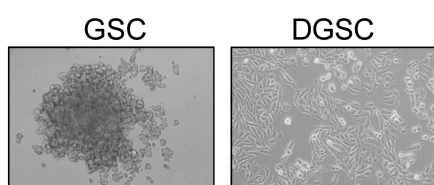

Supplement: Supplementary file 1 [file cells-14-00772-s001.zip › cells-3521342-FigureS2.pdf]
